# Early Sedation Depth and Clinical Outcomes in Mechanically Ventilated Patients with Sepsis

**Published:** 2018-06

**Authors:** Heng FAN, Min SUN, Jian-Hua ZHU

**Affiliations:** Dept. of Intensive Care Unit, Ningbo First Hospital, Ningbo 315000, China

## Dear Editor-in-Chief

The clinical outcomes of mechanically ventilated patients with sepsis are determined by management decisions that made during the initial ventilation including the choice of sedation analgesia agents and the depth of sedation (1, 2). The short-term outcomes of septic adults are plausible associated with the initial sedation depth (3). However, most sedation studies involved patients after 48 h of initial mechanical ventilation, and these patients used different sedation depth that lead to unclear impact on outcomes.

The aims of our study were to investigate the relationship between the early (within 48 h) sedation depth and clinical outcomes of mechanically ventilated patients with sepsis. This prospective longitudinal observational cohort study was conducted in Ningbo First Hospital in China from Jan 2015 to Dec 2016. Inclusion criteria: 1) Age more than 18-year-old and diagnosed as sepsis (4); 2) Mechanical ventilation continuous time more than 48 h; 3) Patients were received dexmedetomidine as sedative within 48 h.

Special definitions: 1. Light sedation was defined as a median Richmond Agitation and Sedation Scale (RASS) range of −2 to +2 and deeply sedation was RASS range of −5 to −3; 2. The confusion assessment method for intensive care (CAM-ICU) score positive was defined as delirious.

This study was approved by ethics committee of Ningbo First Hospital, and the informed consents were obtained from each patient before this study.

Statistical analysis was performed using SPSS 16.0 (SPSS IBM Mandalay Bay, Las Vegas, USA). Multiple linear regressions were used to calculate APACHE II scores, delirium-free days, ventilator-free days, and length of ICU stay. Kaplan-Meier survival curves with a log-rank test were created for 28-day ICU mortality.

**Fig. 1:**
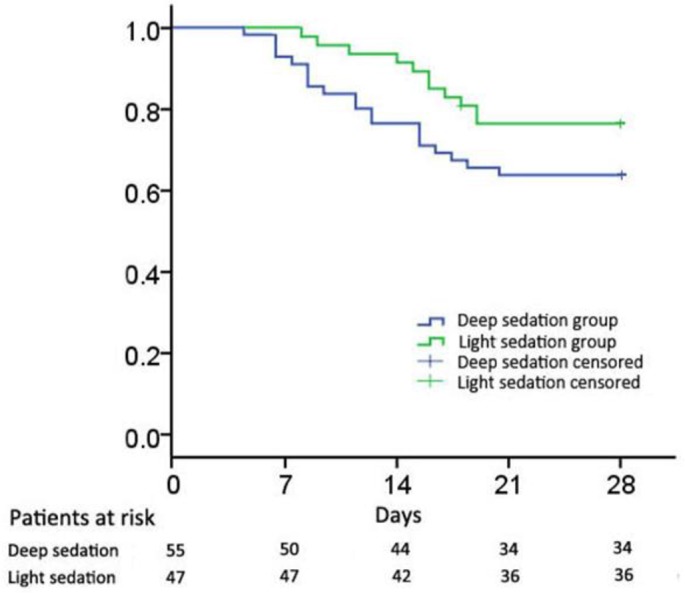
Kaplan-Meier curves for 28-day mortality (log-rank *P*= 0.001)

Overall, 102 septic patients were enrolled and main came from emergency department 35 (34.3%) and the general ward 67 (65.7%) admitted after surgery. Of all these patients, 55 (53.9%) were in deep sedation, 47 (46.1%) were in light sedation. The baseline information of septic patients who received early deep sedation and early light sedation is shown in Table 1. Multivariable analysis showed septic patients with deep sedation had more APACHE II scores [95% CI: 2.87 (1.17 to 4.57)], delirium-free days [1.08 (0.34 to 1.82)], ICU length of stay [3.14 (1.32 to 4.96)], and more ventilator-free days [2.67 (1.10 to 4.24)] than septic patients with light sedation. After adjusting for relevant covariates, the deep sedation group had a greater impact on septic patients compared to those patients with light sedation in terms of APACHE II scores (*P*= 0.003), delirium-free days (*P*= 0.019), ICU length of stay (*P*= 0.010) and ventilator-free days (*P*=0.002). Kaplan-Meier analysis indicated that septic patients with early deep sedation had a significantly higher 28-day mortality (log-rank *P* = 0.001; Fig. 1).

**Table 1:** Demographics and clinical characteristics of septic patients

***Characteristics***	***Deep sedation***	***Light sedation***	***P-value***
N	55	47	
Age, years (IQR)	59 (46–69)	64 (47–73)	0.362
Male gender, n (%)	47 (85.5%)	39 (82.3%)	0.476
APACHE II score, mean (SD)	27.6±4.2	24.7±4.5	0.137
Ventilator-free days, mean (SD)	7.9±4.4	5.2±3.7	0.002
Length of ICU stay, mean (SD)	8.2±3.7	6.9±2.7	0.001
Hospital length of stay, mean (SD)	17.4±5.9	14.2±6.1	0.001
28-day mortality, n (%)	21 (38.2%)	11 (23.4%)	0.001

Early light sedation used dexmedetomidine as sedative may improve the clinical outcomes in septic patients, and a suitable sedation depth as a candidate for deliver interventions is supposed to be well designed within 48 h of initial mechanical ventilation.

## References

[1] Mark van den Boogaard M Schoonhoven L Evers AW (2012). Delirium in critically ill patients: impact on long-term health-related quality of life and cognitive functioning. Crit Care Med, 40(1):112–118. 21926597 10.1097/CCM.0b013e31822e9fc9

[2] Shehabi Y Botha JA Boyle MS (2008). Sedation and delirium in the intensive care unit: an Australian and New Zealand perspective. Anaesth Intensive Care, 36(4):570–578. 18714628 10.1177/0310057X0803600423

[3] Reschreiter H Maiden M Kapila A (2008). Sedation practice in the intensive care unit: a UK national survey. Crit Care, 12(6): R152. 19046459 10.1186/cc7141 PMC2646317

[4] Anonymous (1992). The American College of Chest Physicians/Society of Critical Care Medicine Consensus Conference: definitions for sepsis and organ failure and guidelines for the use of innovative therapies in sepsis. Crit Care Med, 20(6):864–874. 1597042

